# Glycopyrrolate in comparison to hyoscine hydrobromide and placebo in the treatment of hypersalivation induced by clozapine (GOTHIC1): study protocol for a randomised controlled feasibility study

**DOI:** 10.1186/s13063-016-1678-5

**Published:** 2016-11-21

**Authors:** Inti Qurashi, Simon Chu, Nusrat Husain, Richard J. Drake, Imran Chaudhry, J. F. W. Deakin

**Affiliations:** 1 Ashworth Research Centre, Mersey Care NHS Foundation Trust, Liverpool, UK; 2 School of Psychology, University of Central Lancashire, Preston, UK; 3 Institute of Mind, Brain and Behaviour, University of Manchester, Manchester, UK

**Keywords:** Randomised trial, Clozapine, Sialorrhea, Glycopyrrolate, Hyoscine, Feasibility, Adult

## Abstract

**Background:**

Clozapine is the only medication licensed for the treatment of resistant schizophrenia in the UK. Although efficacious, a common and unpopular side effect of clozapine treatment is clozapine-induced hypersalivation (CIH), which can contribute to non-adherence. The standard treatment for CIH in the UK is hyoscine hydrobromide but this may aggravate cognitive deficits in patients with schizophrenia while glycopyrrolate may be an effective alternative with a more tolerable side effect profile. There is currently no convincing evidence for hyoscine, or any other medication, as an effective treatment for CIH.

**Methods/design:**

This is a multicentre randomised, double-blind, placebo-controlled feasibility study of glycopyrronium bromide (glycopyrrolate) and hyoscine hydrobromide (hyoscine) in patients with clozapine-induced hypersalivation. We aim to recruit 42 patients who have been prescribed clozapine and are experiencing hypersalivation, and randomise them to one of three study arms (either hyoscine, glycopyrrolate or placebo). The primary outcome measures will be the participant recruitment and attrition rates, and the secondary outcome will be the metrics of the daytime hypersalivation measure. After a 1-week washout period (discontinuing CIH medication, if any), there will be a 4-week treatment period where participants will be titrated up to the maximum tolerated dose of hyoscine, glycopyrrolate or placebo. Measurements of daytime salivation, nocturnal salivation, cognition and side effects will be taken during home visits in week 2 and week 5. Information on salivation and side effects will also be taken through telephone calls in week 3 and week 4. To gather information on the experience of study participants, exit interviews will also be requested with all participants who drop out of the study and a sample of participants who complete the study.

**Discussion:**

There is currently no convincing evidence for hyoscine, or any other medication, as an effective treatment for CIH. There is promising evidence that glycopyrrolate may be more successful in the treatment of CIH causing fewer cognitive side effects. We propose to conduct a randomised placebo-controlled feasibility study of glycopyrrolate and hyoscine in the treatment of clozapine-induced hypersalivation to inform the design of a future efficacy trial.

**Trial registration:**

Clinicaltrials.gov NCT02613494, 23 November 2015.

**Electronic supplementary material:**

The online version of this article (doi:10.1186/s13063-016-1678-5) contains supplementary material, which is available to authorized users.

## Background

### Study rationale

Clozapine is the only medication licensed for the treatment of resistant schizophrenia in the UK. Approximately 20,000 patients are prescribed clozapine in the UK and, in almost all cases, it is a lifelong prescription. Clozapine-induced hypersalivation (CIH) is a common side effect of clozapine treatment and occurs in up to 80% of patients [1, 2]. CIH may be the source of profound embarrassment and social stigma, lowering self-esteem, increasing social isolation and ultimately exacerbating psychological problems. CIH may also cause inflammation of the salivary glands, salivary calculi, and skin infections [2, 3]. Sleep quality may also be adversely affected, with some patients describing the sensation of choking on excessive saliva at night [4]. There are accompanying practical and financial difficulties (e.g. the regular disposal of soaked pillows and clothing) and a recent survey of clozapine patients showed that hypersalivation is the most undesirable side effect of clozapine treatment [5]. Crucially, adverse side effects may eventually lead to patients discontinuing clozapine treatment [6], considerably increasing the risk of relapse and hospitalisation. The effective treatment of CIH is therefore vital to patient experience and continued wellbeing.

### Current treatment and limitations

Because CIH is not dose-related, a reduction in clozapine dose is not an effective solution and non-pharmacological interventions such as sugarless gum also have very little effect [7]. As a result, CIH is invariably treated with medication. In the UK, the most commonly used medication for CIH is hyoscine hydrobromide (‘hyoscine’), an anti-muscarinic licensed as a prophylactic for motion sickness and as a pre-operative drug to dry secretions. It is readily absorbed from the gastrointestinal tract with an effective bioavailability, for motion sickness, achieved 30 minutes after oral administration. Hyoscine can cause a wide range of side effects, most commonly drowsiness, dizziness and constipation (which can be a lethal side effect of clozapine treatment). In addition, hyoscine crosses the blood-brain barrier, so may cause or exacerbate cognitive deficits, including impairments of visual and verbal memory [8]. This is clinically significant, as cognitive deficits in schizophrenia are common and associated with poor long-term outcomes.

Literature reviews have noted that the majority of studies in CIH provide no convincing evidence for any drug, including hyoscine, as an effective treatment [9]. A Cochrane review of the evidence base for the efficacy of pharmacological treatments for CIH identified 15 randomised controlled trials (RCTs) in the literature, with 14 of these studies conducted in China, significantly limiting their generalizability [10]. The reviewers noted a number of significant problems with study design and data reporting, including the use of cross-over study designs so treatment carry-over effects could not be excluded; the use of unvalidated rating scales to measure hypersalivation; few reports of adverse effects; no reports on effects on general functioning or quality of life; poor quality of data reporting; and none of the studies included hyoscine. The Cochrane review concluded, ‘there are currently insufficient data to confidently inform clinical practice, limitations of these studies are plentiful and the risk of bias high’ and ‘it seems reasonable to study safe interventions for which there is a rationale’ [10].

### Alternative treatment candidate

There is emerging evidence for glycopyrronium bromide (‘glycopyrrolate’), for the treatment of CIH that may be more efficacious, more tolerable and may cause less cognitive impairment. Glycopyrrolate is an anti-muscarinic with weak blood-brain-barrier penetration [11] and is used widely in the UK as a pre-anaesthetic agent because of its ability to decrease salivary production and gastric acid. It is commonly prescribed by paediatricians in the treatment of drooling in children with neurodevelopmental disorders [12]. It is reported to be well tolerated and weak penetration of the blood-brain barrier suggests that side effects involving cognitive impairment are less likely to emerge. In trials of children and young people with a neurological condition the dosage of glycopyrrolate is titrated and dependent on weight, up to a maximum of 3 mg per dose given three times daily. In the only RCT of glycopyrrolate for CIH glycopyrrolate was dosed at 1 mg twice daily. After oral ingestion peak effect is seen at approximately 60 minutes post ingestion.

Three placebo-controlled RCTs of glycopyrrolate (two in children with neurological disorders, one in adults with Parkinson’s disease) have shown significant reductions in excess salivation as a result of glycopyrrolate treatment [13–15]. However the aetiology of drooling in children is likely to be different from drug-induced hypersalivation. Oral preparations of glycopyrrolate are not licensed in the UK for the treatment of CIH, but case reports have described the efficacy of glycopyrrolate in the treatment of CIH [16] and a small (n = 13) double-blind, randomised crossover study of glycopyrrolate and biperiden (a centrally acting anticholinergic similar to hyoscine) in CIH reported a significantly greater reduction in hypersalivation scores in patients receiving glycopyrrolate than those receiving biperiden [17]. Furthermore, participants receiving biperiden showed significantly reduced cognitive functioning in comparison to those receiving glycopyrrolate. The side effects of glycopyrrolate include dry mouth, vomiting, constipation, urinary retention, transient bradycardia, photophobia, flushing of skin and nasal congestion [18]. In one paediatric study, 20% of children who had been administered glycopyrrolate withdrew from the study owing to adverse side effects [13]. However, in the psychiatric population it appears to be well tolerated [17].

### The present study

Several parameters require clarification before a full efficacy trial may proceed. First, whether patients currently undergoing clozapine treatment would be willing to join a randomised placebo-controlled trial (RCT) of hypersalivation medications, and if they are willing to be recruited, whether they will remain in the trial. A secondary issue is there is very little data on the parameters of the daytime hypersalivation measure, the Drooling Rating Scale (DRS), and preliminary work is needed to determine the standard deviation of this measure to inform a sample size calculation for a future RCT.

### Summary

CIH is a common and profoundly debilitating side effect of clozapine treatment that may contribute to non-adherence to treatment [6]. Owing to a poor evidence base and the absence of a viable alternative, clinicians prescribe hyoscine, which may cause impairments in cognition, which in turn may worsen short- and long-term clinical outcomes. There is promising evidence that glycopyrrolate may be more successful in the treatment of CIH causing fewer cognitive side effects. There is a strong rationale for developing a study to compare these medications against each other and we propose to conduct a randomised placebo-controlled feasibility study of glycopyrrolate and hyoscine in the treatment of clozapine-induced hypersalivation to inform the design of a future efficacy trial.

## Methods and design

### Primary objective

To assess the feasibility of recruiting community patients from different centres, the feasibility study will:

- Ascertain whether the study design is acceptable to participants, including randomisation and use of telephone interviews.
- Ascertain whether the interventions are acceptable to participants and indicate likely attrition rates and tolerability.

The primary outcome measures will be participant recruitment and attrition rates.

### Secondary objective

Ascertain the standard deviation of the daytime hypersalivation measure to estimate the required sample size for a future efficacy RCT.

### Study design

This is a multicentre randomised, double-blind, placebo-controlled feasibility study of glycopyrrolate and hyoscine in patients with clozapine-induced hypersalivation. We will recruit 42 patients who have been prescribed clozapine and are experiencing hypersalivation.

**Fig. 1:**
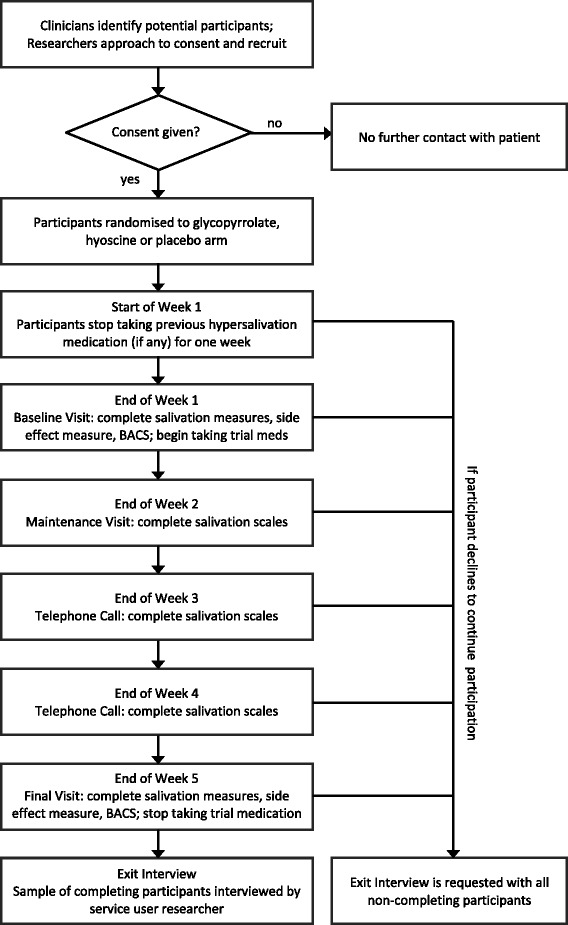
Study flow chart for GOTHIC1

Eligible participants will be recruited to a 5-week study consisting of a 1-week washout period followed by a 4-week treatment period. Participants will be randomised on a 1:1:1 basis to one of three study arms (hyoscine, glycopyrrolate or placebo). Self-report measures of salivation and side effects will be taken weekly and cognition will be assessed at the beginning and end of the study. See Fig. 1 for an overview of the study and Additional file 1 for the Standard Protocol Items: Recommendations for Interventional Trials (SPIRIT) checklist.

### Participant population

Participants will be capacitous, consenting in-patients and community patients recruited from Mersey Care NHS Trust (MCT) and Lancashire Care NHS Foundation Trust (LCFT).

### Inclusion criteria

A participant will qualify for inclusion if he/she:

- has been prescribed clozapine for a minimum of 3 months.
- is experiencing hypersalivation (minimum score of 4 on the Drooling Rating Scale).
- is aged between 18 and 65 years and English speaking.
- is capable of understanding the information given and can provide informed consent prior to study specific procedures.

### Exclusion criteria

- Medical conditions that could influence hypersalivation (e.g. idiopathic Parkinson’s disease).
- Neurological conditions that could affect cognitive functioning during the course of the study (e.g. unstable epilepsy).
- History of an allergic reaction to hyoscine hydrobromide or glycopyrrolate.
- Any of the following contraindications to hyoscine or glycopyrrolate as stated in the British National Formulary: prostatic enlargement, myasthenia gravis, pyloric stenosis, paralytic ileus, toxic megacolon.
- Any of the following cautions to hyoscine or glycopyrrolate as stated in the British National Formulary e.g. closed angle glaucoma, chronic heart failure, chronic lung disease, untreated stomach ulcer, ulcerative colitis, significant liver problems, significant kidney disease, Down’s syndrome, persistent untreated tachycardia, overactive thyroid gland.
- Current prescription for potassium chloride, digoxin, cimetidine, indacaterol, amantadine, atenolol, levodopa or medications that, in the view of the trial pharmacist, have a significant anticholinergic profile.
- A woman of childbearing potential who has tested negative for pregnancy, unable or unwilling to use contraception during the study.
- Participation in another therapeutic study within the preceding 12 weeks or use of other investigational drugs or agents.
- Active suicidal ideation.
- Inpatient on a mental health ward.

### Identification, recruitment and consenting of participants

Participants will be recruited from outpatient clozapine clinics from two NHS mental health trusts. R&D approval will be obtained from each mental health trust prior to start of the study. The study protocol and information sheets will be provided to clinical staff at each participating site who will be asked to identify potential participants who fulfil inclusion/exclusion criteria. Clinical staff will approach potential patients and seek their agreement for research staff to approach them about participation in the study.

After permission has been obtained to approach a potential participant, a researcher will write to them to send study information. The researcher will subsequently contact the patient by telephone and enquire if they are interested in participation. If the patient provisionally agrees, an appointment will be made for the researcher to visit the participant (either at the participant’s home or at their next clinic appointment), in order to answer any further questions the participant may have and to obtain written informed consent.

Following recruitment and consent, the Responsible Clinician and the participant’s GP will be informed in writing. The original, signed copy of the consent form will be retained in the Investigator site file with a copy in clinical notes and a copy provided to the participant. If new safety information results in significant changes in the risk/benefit assessment, the Participant Information Sheet and associated consent form will be reviewed and updated if necessary. If the Participant Information Sheet and consent form are updated, all participants (including those already recruited), will be informed of the new information, given a copy of the revised documents and asked to re-consent to continue in the trial. A screening log will be maintained for all individuals that are offered trial participation and reasons for non-recruitment and screen failures will be recorded.

### Randomisation and blinding

Randomisation will be coordinated by the Manchester Academic Health Sciences Centre Trials Coordination Unit (MAHSC-CTU). After participants have given written consent to participation, a researcher will contact MAHSC-CTU for allocation to one of the three arms of the study on a 1:1:1 basis. The three arms are:

- Hyoscine (arm A – treatment)
- Glycopyrrolate (arm B – treatment)
- Placebo (arm C – control)

Neither the participant nor the researchers will be aware of the arm to which each participant has been allocated and details of the allocation will remain concealed from the research team until after data lock. Blinding of participants and the research team to allocation status will be assured by identical capsule appearance and identical labelling on trial medication (apart from labels identifying the patient). Label randomisation was achieved by a computer-generated list compiled by a statistician at MAHSC-CTU for 42 allocations in two blocks of 21 and supplied to the pharmaceutical company responsible for packaging and labelling of the bottles. In the event a patient develops any side effects where drug unblinding is required, the treating physician will be made aware of the study drug, possible side effects and make the appropriate decision whether to continue or discontinue the drug. The safety and wellbeing of the patient will be paramount at all times.

### Participant withdrawal and replacement

Participants who are removed from the study due to adverse events will be treated and followed according to accepted medical practice. The research team may withdraw a participant from trial because of:

- Adverse event or serious adverse event
- Withdrawal of consent
- Persistent non-compliance with the study protocol
- Sponsor’s decision to terminate the study
- Withdrawal by the Investigator for clinical reasons not related to the study drugs
- Pregnancy
- Symptomatic deterioration including patients who experience rapid deterioration before completion of the protocol treatment.

If a patient withdraws or is withdrawn from the study, it is not the intention to replace them.

### Study drug dosage

#### Hyoscine

The dosing schedule for hyoscine hydrobromide during the 5-week study period is: week 1 - washout period (discontinue existing medication for CIH, if any); week 2 - 300 micrograms twice daily; weeks 3, 4 and 5 - 300 micrograms three times daily. This is within the dose range recommended in the British National Formulary (BNF) (off-licence use for hypersalivation), the Maudsley prescribing guidelines [19] for the treatment of CIH and is consistent with current prescribing practice.

#### Glycopyrrolate

The dosing schedule for glycopyrronium bromide during the 5-week study period will be: week 1 - washout period (discontinue existing medication for CIH, if any); week 2 - 1 milligram twice daily; weeks 3, 4 and 5 - 1 milligram three times daily.

#### Drug administration and accountability

Study drugs and placebo will be dispensed from the trial pharmacy and delivered to participants by the research team. The trial pharmacy will carry out study drug dispensing procedures according to relevant standards, local guidelines and in accordance with Good Clinical Practice guidelines. Participants may receive concomitant therapy deemed to provide adequate care at the treating clinicians’ discretion. The correspondence to the treating mental health clinician and general practitioner following participant recruitment will advise that the study pharmacist is contacted if concomitant medication is prescribed to avoid the prescription of drugs with a significant anticholinergic profile.

The use of experimental drugs is not permitted until at least 14 days after the completion of the study. All medications or other treatments taken by the participant during the study (including those initiated prior to the start of the study) must be recorded in the patient’s clinical notes.

#### Drug compliance and management

Administration of hyoscine, glycopyrrolate or placebo will be recorded in the Case Report Form (CRF). At each weekly contact by the researcher, the participant will be asked about how many doses were taken or omitted. Omission of drug doses will be recorded in the CRF.

Participants will remain under the care of their clinical team during the period of the study. In the event of an accidental or intentional overdose participants will be advised to seek urgent medical attention in casualty departments and emergency unblinding will be undertaken.

### Study procedures

#### Data collection procedures

There will be three visits in total by a researcher and there may also be one further visit to conduct an exit interview if a participant consents to this. A baseline visit will take place at the end of the week 1 washout period, where a researcher will visit the participant at home to deliver trial medication for week 2 and administer all assessment measures. The maintenance visit will take place at the end of week 2, where a researcher will visit the participant at home to deliver trial medication for weeks 3–5 and administer hypersalivation scales and the side effects scale. The final visit will take place at the end of week 5 where a researcher will visit the participant at home to administer all assessment measures again. Appointments will be made on each occasion and researchers will remind participants of forthcoming appointments via telephone call. If a participant is not available when the researcher arrives for any visit, another appointment will be made as soon as possible.

An important aim is to explore the feasibility of collecting information on hypersalivation and side effects using weekly telephone calls assessing. At the end of week 3 and week 4, a researcher will telephone the participant at a prearranged time and administer hypersalivation scales and the side effects scale.

Researchers will request an exit interview with all participants who drop out of the study and a number of participants who complete the study until 12 interviews have been conducted. Interviews will take place at the participant’s home at their convenience Two researchers (one of whom will also be a service user) will conduct a 20-minute interview to explore the participant’s experience of taking part in the study, exploring the acceptability of the study methods and seeking advice on how study participation experience could be improved. Of particular interest will be the views of participants who did not complete the study and in particular their reasons for dropping out, so that this may inform the design of the future RCT with respect to reducing attrition rates.

A review of each participant’s case notes will be undertaken on three occasions (consent, baseline and final visit) to obtain information on concurrent medication prescribed and record any adverse or side effects not directly reported by the participant.

#### Assessment measures

##### Drooling Rating Scale (DRS)

The DRS [20] is a two-item scale comprising drooling severity and frequency assessments that combine to form a score ranging from 2 to 9. Whilst it has not been validated and its metrics (standard deviation, mean, sensitivity to change) are unknown in a UK CIH population, it has good face validity and has been used in published research on paediatric hypersalivation [17, 21]. A feasibility aim is to establish its metrics in a UK CIH population.

##### Nocturnal Hypersalivation Rating Scale (NHRS)

The NHRS [22] is a validated single-item 5-point self-report scale for measuring the degree of nocturnal salivation that a respondent experiences. The NHRS is the only scale specifically mentioned in the Cochrane review for treatments for CIH [10] that is recommended for inclusion in future studies of the efficacy of CIH interventions.

##### Brief Assessment of Cognition in Schizophrenia (BACS)

The BACS [23] comprises a short battery of tests devised for easy administration and scoring which assess the extent of cognitive impairment in schizophrenia. The battery includes brief assessments of executive functions, verbal fluency, attention, verbal memory, working memory and motor speed and requires approximately 30 minutes to complete.

##### Liverpool University Neuroleptic Side Effect Rating Scale (LUNSERS)

LUNSERS [24] is a 51-item checklist of side effects which assesses on a 5-point scale the degree to which respondents have experienced that side effect in the last month. It shows good reliability and validity, correlating well with other clinician-administered side effect scales. A modified LUNSERS will be used to assess side effects in the previous week (rather than month).

#### Sample size considerations

Our intended sample size is based on recruiting sufficient numbers to fulfil the feasibility aims of the study. The intention is to recruit 14 patients to each of three study arms because recruitment for a total sample of 42 will allow a good estimate of recruitment and attrition rates and allows a conservative estimated recruitment rate of one participant per week over an 11-month period. We estimate an attrition rate of 20% (which is substantially higher than in previous studies) which implies nine dropouts and this allows clear differentiation from one criterion (40% drop-out, n = 17) for future progression to a full efficacy trial, with more than 80% power to detect a difference of this size with alpha at 0.25 (one-tailed). This is consistent with the relaxed power and alpha criteria suitable for an early phase study of this type [25, 26] specifically the alpha 0.25 criterion advocated by Schoenfeld [27]. As a secondary consideration, this sample size also gives more than 80% power to detect a difference between arms of effect size 0.5 [17] with alpha 0.1 (two-tailed), assuming correlation between measures at each of five time points of r > = 0.5. Furthermore, 14 in each arm will be sufficient to gain an indication of the metrics (standard deviation (SD), mean, sensitivity to change) for the putative primary outcome measure (DRS) in placebo and active treatment arms recruited from this population for a future efficacy study.

#### Safety reporting procedures

All adverse events (AEs) suspected of having a causal relationship to either study drug will be captured in the participant’s clinical notes and CRF from the start of treatment (week 2) until the end of the participant’s involvement in the study. Serious adverse events (SAEs) will also be reported to the MAHSC-CTU within 24 hours of observing or learning about the event. The study manager will liaise with the Chief Investigator (CI) to evaluate the event for seriousness, causality and expectedness. All SAEs will be followed up until resolution and the participating site must provide follow-up reports if the SAE has not resolved at the time the initial report was submitted.

#### Trial management and quality assurance

The study is managed through the Trial Management Group (TMG) which includes those individuals responsible for the day-to-day management of the study including the CI, co-investigators and the study manager. The TMG has operational responsibility for the conduct of the study including monitoring overall progress and taking appropriate action to safeguard participants and the quality of the study. The TMG meets at least quarterly once the study is actively recruiting. The study manager and CI will ensure that all relevant issues and actions discussed during the meeting are followed up and resolved. At least annually, the TMG will be extended to invite an independent chair (not involved directly in the study) and additional independent members, a service user representative, and the assembly will be conducted as a Trial Steering Committee (TSC) meeting. The TSC takes responsibility for the scientific integrity of the study, the scientific validity of the study protocol, assessment of the study quality and conduct as well as for the scientific quality of the final study report. Decisions about the continuation or termination of the study or substantial amendments to the protocol are the responsibility of the TSC. The Principal Investigators at the participating sites are responsible for the day-to-day running of the study at their site.

TSC meetings will be organised by the CI via the study manager. Minutes will be taken at TSC meetings and copies of the minutes will be filed in the Trial Master File. The study manager and CI will ensure that all relevant issues and actions discussed during the meeting are followed up and resolved. The committee’s terms of reference, roles and responsibilities will be defined in a charter issued by the MAHSC-CTU.

No Data Monitoring Committee will be required for this study because the outcome measures do not require the review of study data.

## Discussion

We aim to conduct a large-scale randomised placebo-controlled trial of hyoscine and glycopyrrolate in the treatment of clozapine-induced hypersalivation. As a step towards this, the present feasibility study will provide information on recruitment, tolerability and attrition, acceptability of the study design and the views of study participants who complete and drop out of the study to inform both the viability and design of the future efficacy trial.

### Trial sponsor

The Sponsor of the study is Mersey Care NHS Foundation Trust. The Sponsor has no part in study design, nor the collection, management and analysis of data, nor the decisions to submit reports for publication.

### Trial status

This clinical trial was registered on 23 November 2015 (ClinicalTrials.gov identifier: NCT02613494) and will begin recruitment in January 2017. The estimated study completion date is September 2017.

## Additional file

Additional file 1: Standard Protocol Items: Recommendations for Interventional Trials (SPIRIT) checklist. (DOC 120 kb)

## Abbreviations

AE: adverse event
BACS: Brief Assessment of Cognition in Schizophrenia
BNF: British National Formulary
CI: Chief Investigator
CIH: clozapine-induced hypersalivation
CRF: Case Report Form
DRS: Drooling Rating Scale
LCFT: Lancashire Care NHS Foundation Trust
LUNSERS: Liverpool University Neuroleptic Side Effect Rating Scale
MAHSC-CTU: Manchester Academic Health Science Centre Trials Coordination Unit
MCT: Mersey Care NHS Trust
NHRS: Nocturnal Hypersalivation Rating Scale
RCT: randomised controlled trial
REC: Research Ethics Committee
SAE: serious adverse event
SD: standard deviation
TMG: Trial Management Group
TSC: Trial Steering Committee

## References

[1] Waserman J, Criollo M (2000). Subjective experiences of clozapine treatment by patients with chronic schizophrenia. Psychiatr Serv.

[2] Safferman A, Lieberman JA, Kane JM (1991). Update on the clinical efficacy and side-effects of clozapine. Schizophr Bull.

[3] Brodkin ES, Pelton GH, Price LH (1996). Treatment of clozapine-induced parotid gland swelling. Am J Psychiatry.

[4] Hinkes R, Quesada T, Currier MB (1996). Aspiration pneumonia possibly secondary to clozapine-induced sialorrhea. J Clin Psychopharmacol.

[5] Qurashi I, Stephenson P, Chu S, Duffy C, Husain N, Chaudhry I (2015). An evaluation of subjective experiences, effects and overall satisfaction with clozapine treatment in a UK forensic service. Ther Adv Psychopharmacol.

[6] Krivoy A, Malka L, Fischel T, Weizman A, Valevski A (2011). Predictors of clozapine discontinuation in patients with schizophrenia. Int Clin Psychopharmacol.

[7] UK Medicines Information for NHS healthcare professionals. ‘Q and A 54.4. Drug-induced hypersalivation – what treatment options are available?’ www.awp.nhs.uk/handlers/downloads.ashx?id=6362. Accessed 17 Nov 2016.

[8] Sherman SJ, Atri A, Hasselmo ME, Stern CE, Howard MW (2003). Scopolamine impairs human recognition memory: data and modelling. Behav Neurosci.

[9] Sockalingam S, Shammi C, Remington G (2007). Clozapine-induced hypersalivation: a review of treatment strategies. Can J Psychiatry.

[10] Syed R, Au K, Cahill C, Duggan L, He Y, Udu V, Xia J (2008). Pharmacological interventions for clozapine-induced hypersalivation. Cochrane Database Syst Rev.

[11] Reddihough D, Reid S, Plover C (2011). Evaluation of glycopyrrolate in the treatment of chronic drooling. Degener Neurol Neuromuscul Dis.

[12] Parr JR, Buswell CA, Banerjee KJ, Fairhurst C, Williams J, O’Hare A (2012). Management of drooling: a survey of UK clinicians’ clinical practice. Child Care Health Dev.

[13] Mier RJ, Bachrach SJ, Lakin RC, Barker T, Childs J, Moran M (2000). Treatment of sialorrhea with glycopyrrolate: a double-blind, dose-ranging study. Arch Pediatr Adolesc Med.

[14] Zeller R, Davidson J, Lee H, Cavanaugh P (2012). Safety and efficacy of glycopyrrolate oral solution for management of pathological drooling in paediatric patients with cerebral palsy and other neurological conditions. Ther Clin Risk Manage.

[15] Arbouw ME, Movig KL, Koopmann M, Poels PJ, Guchelaar HJ, Egberts TC, Nef C, van Vugt JP (2010). Glycopyrrolate for sialorrhea in Parkinson disease: a randomized, double-blind, crossover trial. Neurology.

[16] Robb AS, Lee RH, Cooper EB, Siedel JV, Nusrat N (2008). Glycopyrrolate for treatment of clozapine-induced sialorrhea in three adolescents. J Child Adolesc Psychopharmacol.

[17] Liang CS, Ho PS, Shen LJ, Lee WK, Yang FW, Chiang KT (2010). Comparison of the efficacy and impact on cognition of glycopyrrolate and biperiden for clozapine-induced sialorrhea in schizophrenic patients: a randomized, double-blind, crossover study. Schizophr Res.

[18] British Medical Association and the Royal Pharmaceutical Society (2011). British National Formulary (62).

[19] Taylor D, Paton C, Kapur S (2015). The Maudsley prescribing guidelines in psychiatry.

[20] Thomas-Stonell N, Greenberg J (1988). Three treatment approaches and clinical factors in the reduction of drooling. Dysphagia.

[21] Evatt ML (2011). Oral glycopyrrolate for the treatment of chronic severe drooling caused by neurological disorders in children. Neuropsychiatr Dis Treat.

[22] Spivak B, Adlersberg S, Rosen L, Gonen N, Mester R, Weizman A (1997). Trihexyphenidyl treatment of clozapine-induced hypersalivation. Int Clin Psychopharmacol.

[23] Keefe RSE, Goldberg TE, Harvey PD, Gold JM, Poe MP, Coughenour L (2004). The brief assessment of cognition in schizophrenia: reliability, sensitivity, and comparison with a standard neurocognitive battery. Schizophr Res.

[24] Day JC, Wood G, Dewey M, Bentall RP (1995). A self-rating scale for measuring neuroleptic side-effects: Validation in a group of schizophrenic patients. Br J Psychiatry.

[25] Moore CG, Carter RE, Nietert PJ, Stewart PW (2011). Recommendations for planning pilot studies in clinical and translational research. Clin Transl Sci.

[26] Julious SA (2005). Sample size of 12 per group rule of thumb for a pilot study. Pharm Stat.

[27] Schoenfeld D (1980). Statistical considerations for pilot studies. Int J Radiat Oncol Biol Phys.

[28] World Health Organization (2005). Handbook for good clinical research practice (GCP): guidance for implementation.

[29] World Medical Association: Declaration of Helsinki. Ethical principles for medical research involving human subjects. www.wma.net/en/30publications/10policies/b3/index.html. Accessed 17 Nov 2016.

